# The tricky effects of TROP2 in lung cancer: from clinical practice back to fundamental investigations

**DOI:** 10.3389/fonc.2025.1638054

**Published:** 2025-08-22

**Authors:** Renwang Liu, Mingbiao Li, Penghu Gao, Xuanguang Li, Zihe Zhang, Jun Chen

**Affiliations:** ^1^ Department of Lung Cancer Surgery, Department of Thoracic Surgery, Tianjin Medical University General Hospital, Tianjin, China; ^2^ Tianjin Key Laboratory of Lung Cancer Metastasis and Tumor Microenvironment, Tianjin Lung Cancer Institute, Tianjin Medical University General Hospital, Tianjin, China

**Keywords:** Trop2, antibody-drug conjugates, lung cancer, bioinformatics analysis, molecular mechanism

## Abstract

TROP2, a transmembrane glycoprotein, is overexpressed and plays pivotal roles in diverse epithelial tumors. The differential expression of TROP2 between cancer and normal tissues offers distinct advantages in developing drugs targeting it. Thus, TROP2-targeted antibody-drug conjugates (ADCs), including datopotamab deruxtecan and sacituzumab govitecan, present considerable efficacy and safety in multiple cancers. However, in lung cancer, the application of TROP2-targeted ADCs has many limitations and challenges. The current clinical trials have not achieved encouraging results yet. Meanwhile, the expression of TROP2 in lung cancer remains ambiguous, let alone its biological effects and underlying mechanism. The complex features and limited research may slow down the development of TROP2-targeted ADCs in lung cancer. Therefore, we comprehensively reviewed the literature on TROP2 in lung cancer, extending back to basic research from clinical trials. We also combined several preliminary bioinformatics analyses in this review and intended to find some research directions on breaking through these limitations.

## 1 Introduction

Lung cancer remains a leading cause of morbidity and mortality among malignancies globally (1, 2). Non-small cell lung cancer (NSCLC) constitutes approximately 85% of all lung cancers, with adenocarcinoma and squamous cell carcinoma being the major subtypes, and small cell lung cancer (SCLC) accounts for about 15% (3–5). Despite the remarkable progress made in lung cancer treatments recently, the existing strategies still have significant limitations (6–8). For example, chemotherapy damages normal tissues due to poor specificity (9, 10); targeted therapy, despite fewer side effects, nearly always develops drug resistance over time (11, 12); and immune checkpoint inhibitors can occasionally trigger immune reactions in vital organs, causing severe adverse events (13, 14). Thus, exploring novel treatment targets and methods is crucial for improving survival and quality of life in lung cancer patients.

TROP2 (trophoblast cell-surface antigen 2), also known as tumor-associated calcium signal transducer 2 (TACSTD2), is a transmembrane glycoprotein widely overexpressed in epithelial-derived tumors (15–17). Studies on various cancers, such as breast, colorectal, and bladder, have shown that TROP2 significantly influences tumor cell proliferation, migration, invasion, and metastasis (18–20). Given its high expression characteristic, drugs targeting TROP2 can precisely target cancer cells, minimizing damage to normal cells and disrupting tumor-related signaling pathways (21–23). Thus, TROP2-targeted antibody-drug conjugates (ADCs), which link cytotoxic drugs to TROP2-specific antibodies and have succeeded in treating these cancers, may offer potential for lung cancer treatment (21, 22, 24).

However, unlike in other tumors, results of current clinical trials on TROP2-targeted ADCs in lung cancer are not promising. The ambiguous differential expression and effects may be ascribable. The unknown underlying regulatory mechanisms also impede the development of TROP2-targeted ADCs.

Thus, we reviewed the recent advances of TROP2-targeted ADCs in the field of lung cancer. We also summarized the basic and translational research on TROP2 in lung cancer, including its links to clinical features, prognostic significance, and molecular mechanisms. We further enrolled preliminary explorations via online bioinformatics tools, aiming to provide potential insights for future research and treatment development.

## 2 TROP2-targeted treatment in lung cancer and its limitations

### 2.1 TROP2-targeted ADCs in lung cancer therapy

ADCs are a novel class of anti-cancer agents (25–27), consisting primarily of monoclonal antibodies, linkers, and cytotoxic drugs (28–30). The monoclonal antibodies can specifically recognize antigens in cancer, guiding the entire ADC precisely to tumor cells (16, 21, 22). TROP2 is one of the most common antigens; it is significantly overexpressed in a wide range of tumor cells, allowing TROP2-targeted ADCs to show promising efficacy in clinical trials among breast, colorectal, and bladder cancers (18–20).

Theoretically, TROP2-targeted ADCs should also have definite efficacy in lung cancer. For example, by using NSCLC patient-derived xenograft (PDX) models, Daisuke Okajima et al. found that datopotamab deruxtecan (Dato-DXd) specifically binds TROP2, is then internalized by tumor cells and transported to lysosomes through intracellular trafficking mechanisms (21). In lysosomes, the linker is cleaved, releasing the connected cytotoxic drug, a type of topoisomerase I inhibitor, which then induces DNA damage and apoptosis in tumor cells (21). The phase I TROPION-PanTumor01 trial, which assessed TROP2 via immunohistochemistry (IHC) and enrolled patients without a minimum TROP2 expression, showed that in NSCLC patients receiving 6mg/kg, the objective response rate was 26%; median duration of response was 10.5 months; and median progression-free survival (PFS) and overall survival (OS) were 6.9 and 11.4 months, respectively (22). The IMMU-132–01 basket trial also showed that several NSCLC patients achieved remission (31).In SCLC, sacituzumab govitecan (SG) has also shown efficacy in treating metastatic patients (32).

However, the limitations and challenges are evident. Firstly, in most cancers, TROP2 expression predicts responses to TROP2-targeted ADCs. For example, as Shuying Qiu et al. reviewed, the expression of TROP2 may be positively correlated with SG sensitivity in uterine carcinoma and ovarian cancer (33). In breast cancer, TROP2 expression might also enhance SG responsiveness in specific subtypes, as reviewed by Liqin Yao et al (34). Unlike in other cancer types, the efficacy of TROP2-targeted ADCs is not yet established and appears unrelated to TROP2 expression in lung cancer (22, 32). A substantial fraction of patients presenting high TROP2 expression even exhibit complete non-responsiveness to ADCs (22, 31). Outcomes of TROP2-targeted ADCs in lung cancer have also been less encouraging than those in triple-negative breast cancer (TNBC) and metastatic urothelial cancer (mUC) (22, 31, 32). The underlying reason remains unclear. As Toshio Shimizu noted in the discussion, differences in internalization efficiency, the amount of DXd released via lysosomal proteases, and the sensitivity of cancer cells to deruxtecan may all lead to variations in Dato-DXd’s efficacy (22).

Additionally, safety also presents a notable challenge. Prevalent adverse events include interstitial lung disease, pneumonia, infusion-related reactions, oral mucositis, and stomatitis (35). In the TROPION-PanTumor01 trial, 6% of patients developed interstitial lung disease (22). In IMMU-132-01, severe adverse events included febrile neutropenia (4.0%) and diarrhea (2.8%) (31). This trial also reported one treatment-related death from an adverse event of aspiration pneumonia (31). TROP2 is widely expressed across tissues, with the respiratory epithelium being a major site of high expression (17). This may be one of the important reasons for its tendency to cause lung-specific toxicity. Pulmonary function impairment from central lung cancer, extensive pulmonary metastases, or other space-occupying lesions, combined with drug-induced pulmonary toxicity, may disrupt TROP2-targeted ADC therapy and diminish efficacy.

### 2.2 Combination therapy: a potential solution with unknown efficacy

Combining TROP2-targeted ADCs with other drugs may be a promising therapeutic strategy for overcoming the above limitations. In 2023, Melissa L. Abel reported on a Phase I clinical trial, showing that SG combined with berzosertib may improve safety over conventional chemotherapy-based combinations, as no clinically relevant ≥grade 4 adverse events occurred (36). It also resulted in a response in one SCLC patient (transformed from epidermal growth factor receptor (EGFR)-mutant NSCLC), with a partial response lasting 17 weeks (36). This study supports the potential of the combination strategies.

Latest clinical trials on combination therapy are also currently underway. Okamoto I et al. launched a study to evaluate the efficacy of Dato-DXd combined with pembrolizumab ± platinum-based chemotherapy as first-line treatment for advanced NSCLC, named TROPION-Lung07 (no TROP2 expression requirement for enrolled patients; TROP2 detection method not mentioned) (37). Levy BP et al. initiated the TROPION-Lung08 study which similarly had no TROP2 expression requirement for enrollment and did not specify TROP2 detection methods. This study set up a Dato-DXd combined with pembrolizumab group and a pembrolizumab monotherapy group to compare the efficacy of their combined application in specific patients with advanced NSCLC (38). If these trials yield promising results in the future, they could offer a novel therapeutic strategy for lung cancer.

## 3 TROP2 expression in lung cancer and its clinical implications

As discussed above, while TROP2-targeted ADCs show potential in lung cancer treatment, their efficacy remains unconfirmed and challenges persist. Combination therapy may offer a potential solution but remains exploratory. More intriguingly, efficacy shows no correlation with TROP2 expression in either NSCLC or SCLC (22, 32). The identification of specific biomarkers, effects, and mechanisms may represent a critical breakthrough for overcoming limitations in both efficacy and safety. Therefore, this review advocates shifting from clinical observations to basic research to identify potential directions.

### 3.1 Elusive differential expression of TROP2

While TROP2 is significantly overexpressed in most cancer types, including breast cancer (33, 34), its expression in lung cancer tissues remains controversial. Jiang A et al. confirmed significant TROP2 overexpression in lung cancer tissues using a cohort of 87 NSCLC patients (39). M Trerotola et al. similarly reported that TROP2 expression was significantly higher in lung cancer than in normal tissues (40). Inamura K et al. compared different NSCLC subtypes and found that TROP2 protein levels were significantly increased in 64% (172/270) of adenocarcinomas, 75% (150/201) of squamous cell carcinomas, and 18% (21/115) of high-grade neuroendocrine tumors (HGNETs) (41). These studies suggest that TROP2 may be generally upregulated in lung cancer. However, the opposite results in lung adenocarcinoma (LUAD) tissues have also been reported. Its low expression may be related to loss of heterozygosity (LOH) or hypermethylation of CpG islands in its promoter region (42). Different pathological types also exhibit distinct expression patterns. For example, Pak MG et al. compared 164 NSCLC patients and found that TROP2 was expressed significantly higher in squamous cell carcinoma (100/164) than in adenocarcinoma (64/164) (43). We summarized all reported expression patterns of TROP2 in lung cancers and showed them in **Table 1**.

**Table 1:** TROP2 expression in lung cancer and its clinical implications.

Authors	Published years	Differential expression of Trop2 in lung cancer					Association with clinical features and prognosis						
		Pathological type	Sample size	Expression status	Detection methods	Expression level	Age	Gender	Histological grade	TNM staging	LN metastasis	PS score	Prognosis
Kentaro Inamura, et al (41)	2017	AdC, SqCC & HGNET	270 of AdC; 201 of SqCC; 115 of HGNET	High exp. in 64% of AdC, 75% in SqCC and 18% in HGNETs	IHC	Protein	NSS in AdC; N/A in SqCC & HGNETs	High expression in males in AdC; N/A in SqCC & HGNETs	Lower differentiation in AdC; N/A in SqCC & HGNETs	Higher stage in AdC; N/A in SqCC & HGNETs	N/A	N/A	Higher mortality in AdC; NSS in SqCC; lower mortality in HGNETS
Min Gyoung Pak, et al (43)	2012	AdC, SqCC	100 of AdC,64 of SqCC	High exp. in 23.0% of AdC, 64.1% in SqCC	IHC	Protein	NSS	NSS	Lower differentiation in AdC; NSS in SqCC	N/A	N/A	N/A	Lower mortality in AdC; N/A in SqCC
Remi Mito, et al (48)	2020	AdC, SqCC	231 of AdC,103 of SqCC	Expressed in all AdC and 92% of SqCC	IHC	Protein	NSS	NSS	NSS	NSS	N/A	N/A	Higher mortality in AdC (Especially in non-EGFR mutation and lower differentiation); NSS in SqCC
Zanhua Li, et al (47)	2016	AdC	68	High exp.	IHC & qPCR	Protein & mRNA	NSS	NSS	Higher differentiation	More advanced	Higher metastasis rate	N/A	Higher mortality
Jau-Chen Lin et al (42)	2012	AdC	63	Low exp.	55 by IHC;8 by WB	Protein	NSS	NSS	N/A	NSS	N/A	N/A	N/A
Aigui Jiang, et al (39)	2013	AdC, SqCC	50 of AdC,37 of SqCC	High exp. in 42.0% of AdC and 67.6% in SqCC	IHC	Protein	NSS	NSS	Lower differentiation	More advanced in AdC; NSS in SqCC	Higher metastasis rate in AdC; NSS in SqCC	NSS	Higher mortality in AdC; NSS in SqCC
M Trerotola, et al (40)	2013	Lung cancer	21 for mRNA, 72 for protein level	High exp.	21 by Comparative SAGE; 72 by IHC	Protein & mRNA	N/A						

NSS, No Significant Statistical Significance; N/A, Not Applicable; AdC, Adenocarcinoma; SqCC, Squamous Cell Carcinoma; HGNET, High-Grade Neuroendocrine Tumor; IHC, Immunohistochemistry; LN, Lymph Node; Exp., expression; PS, Performance Status; SAGE, Serial Analysis of Gene Expression.

**Figure 1:**
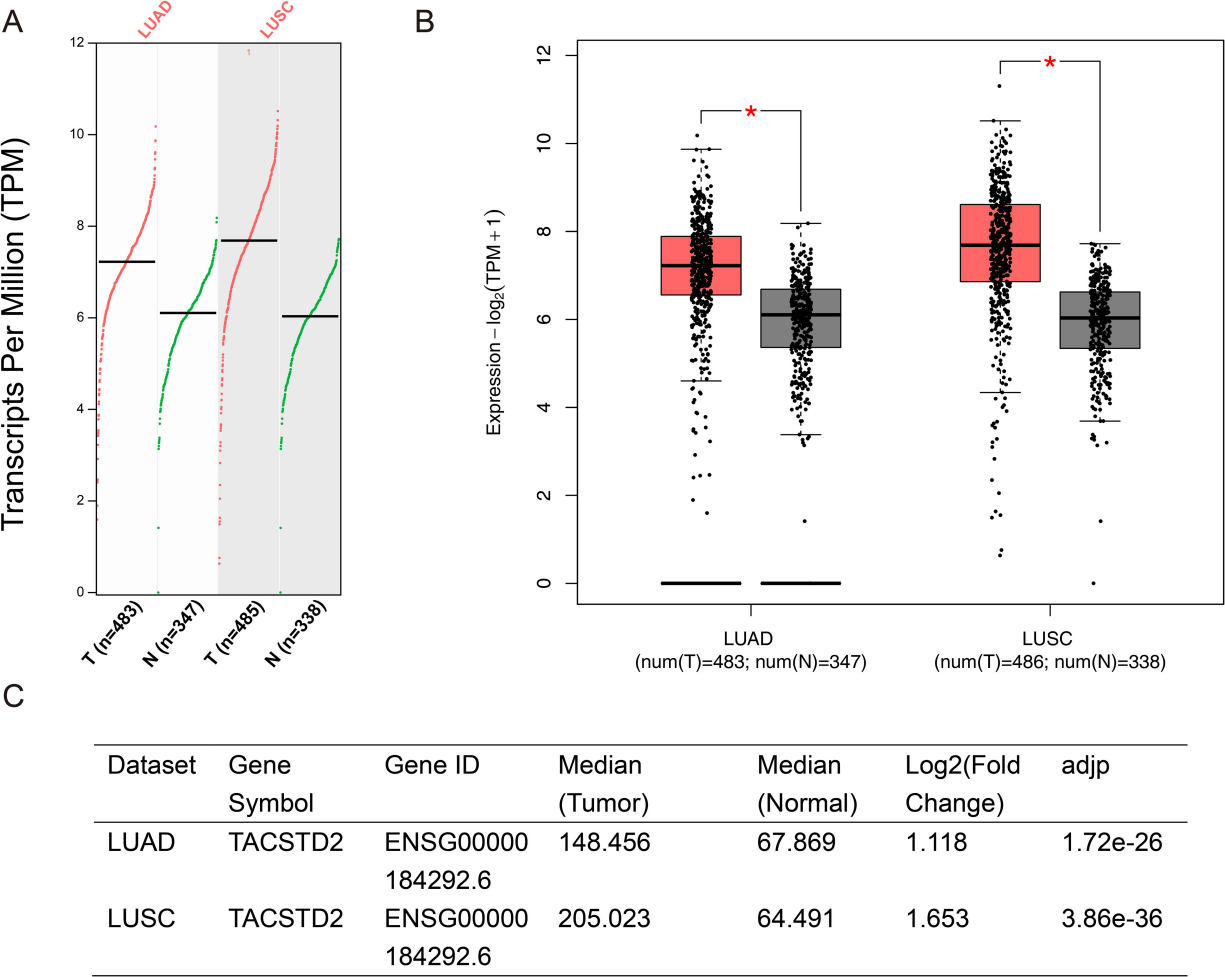
Preliminary Bioinformatics Analysis on Differential Expression of TROP2. The differential expression of TROP2 was analyzed by GEPIA2 (http://gepia2.cancer-pku.cn/#index), and the data were based on the TCGA database (https://portal.gdc.cancer.gov/). TROP2 was significantly overexpressed in tumor tissues compared with normal tissues in both LUAD and LUSC. The scatter plot is shown in **(A)** and the bar chart is shown in **(B)**. The parameter settings were defined as a Log2FC Cutoff of 1 and a q-value Cutoff of 0.01. The ANOVA was selected as the major method. The log2 fold-changes of TROP2 in the tumor compared with normal tissue were 1.12 and 1.65 in LUAD and LUSC, respectively **(C)**.

**Figure 2:**
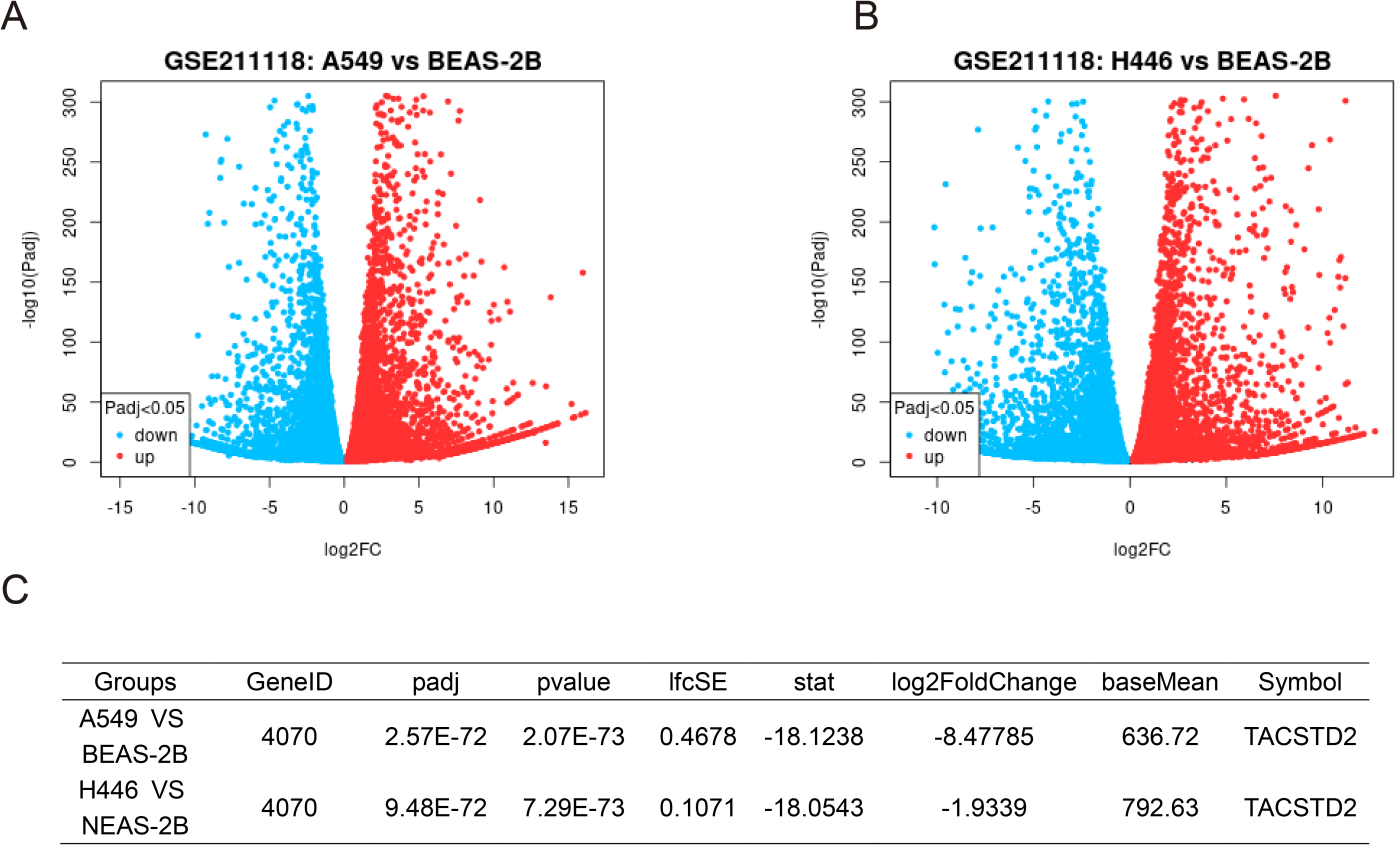
Differential Expression Analysis of GSE211118 Dataset Identified TROP2 Downregulation in Lung Cancer Cell Lines. The GSE21118 dataset includes RNA sequencing data from lung cancer cell lines (A549, H446) and the normal alveolar cell line (BEAS-2B), with three biological replicates before and after treatment. To identify DEGs, we selected the pre-treatment data of these three cell lines and employed the GEO2R online tool for comparative analysis. Using the tool’s default parameters, we generated volcano plots for A549 **(A)** and H446 **(B)**. Notably, TROP2 was significantly downregulated in both cancer cell lines, with log2 fold-changes of -8.48 in A549 and -1.93 in H446 **(C)**.

To further explore these controversial expression patterns, we queried the open-access bioinformatics analysis tool Gene Expression Profiling Interactive Analysis (GEPIA) (http://gepia2.cancer-pku.cn/#index) and analyzed the data from The Cancer Genome Atlas (TCGA) (https://portal.gdc.cancer.gov/) (44). Results showed that TROP2 was significantly overexpressed in both LUAD and lung squamous cell carcinoma (LUSC) (**Figure 1**). We also retrieved RNA sequencing results of lung cancer cells and normal bronchial epithelial cell lines. The sequencing data of GSE211118 were then analyzed by using the online tool GEO2R (45). Notably, in this dataset, we used the pre-treatment data of A549 (a human NSCLC cell line) and H446 (a human SCLC line) as the sequencing results for lung cancer cells, and the pre-treatment data of BEAS-2B (a human normal bronchial epithelial cell line) as normal cell controls. The results showed that TROP2 significantly underexpressed in both A549 and H446 cell lines (**Figure 2**). The differentially expressed genes (DEGs) are listed in **Supplementary Material S1** (statistical results table of DEGs for A549 vs BEAS-2B) and **Supplementary Material S2** (statistical results table of DEGs for H446 vs BEAS-2B). These preliminary bioinformatics analyses thus revealed a striking inconsistency in TROP2 expression patterns between tissues and cell lines. This inconsistency may lead to the reported controversial results discussed above. Other factors including different detection methods and timing, and lack of multi-dimensional analyses may also hinder scholars from clarifying the specific expression pattern of TROP2 in lung cancer.

It should be noted that our bioinformatics analyses have some limitations. Firstly, both the TCGA database and GSE211118 data only provide TROP2 mRNA detection results. In the mechanism of ADCs, the key process involves specific binding to membrane proteins to guide drug delivery. Thus, although a study has shown a significant positive correlation between TROP2 mRNA expression and protein expression in NSCLC (46), the lack of protein-level research may still lead to result bias. Additionally, our preliminary bioinformatics analyses did not explore TROP2 expression in various lung cancer subtypes. Future research at this level may help identify specific subtypes with significant efficacy of TROP2-targeted ADCs.

### 3.2 Association with clinical features and prognosis

TROP2 also shows inconsistent correlations with clinical features in lung cancer (**Table 1**). Pak MG et al. found that TROP2 only correlated significantly with histologic grade in LUAD (43). In contrast, Li Z et al. confirmed that TROP2 was significantly correlated with the TNM stage (P = 0.012), lymph node metastasis (P = 0.038), and histological grade (P = 0.013) (47). In squamous cell carcinoma, TROP2 was also related to the histological grade but not to gender, age, lymph node metastasis, TNM stage, or PS score (39). Peiwen Kuo et al. also found that the expression of TROP2 in NSCLC was not correlated with age, gender and race (46). While, in large cell lung cancer or SCLC, the correlations were rarely reported.

**Figure 3:**
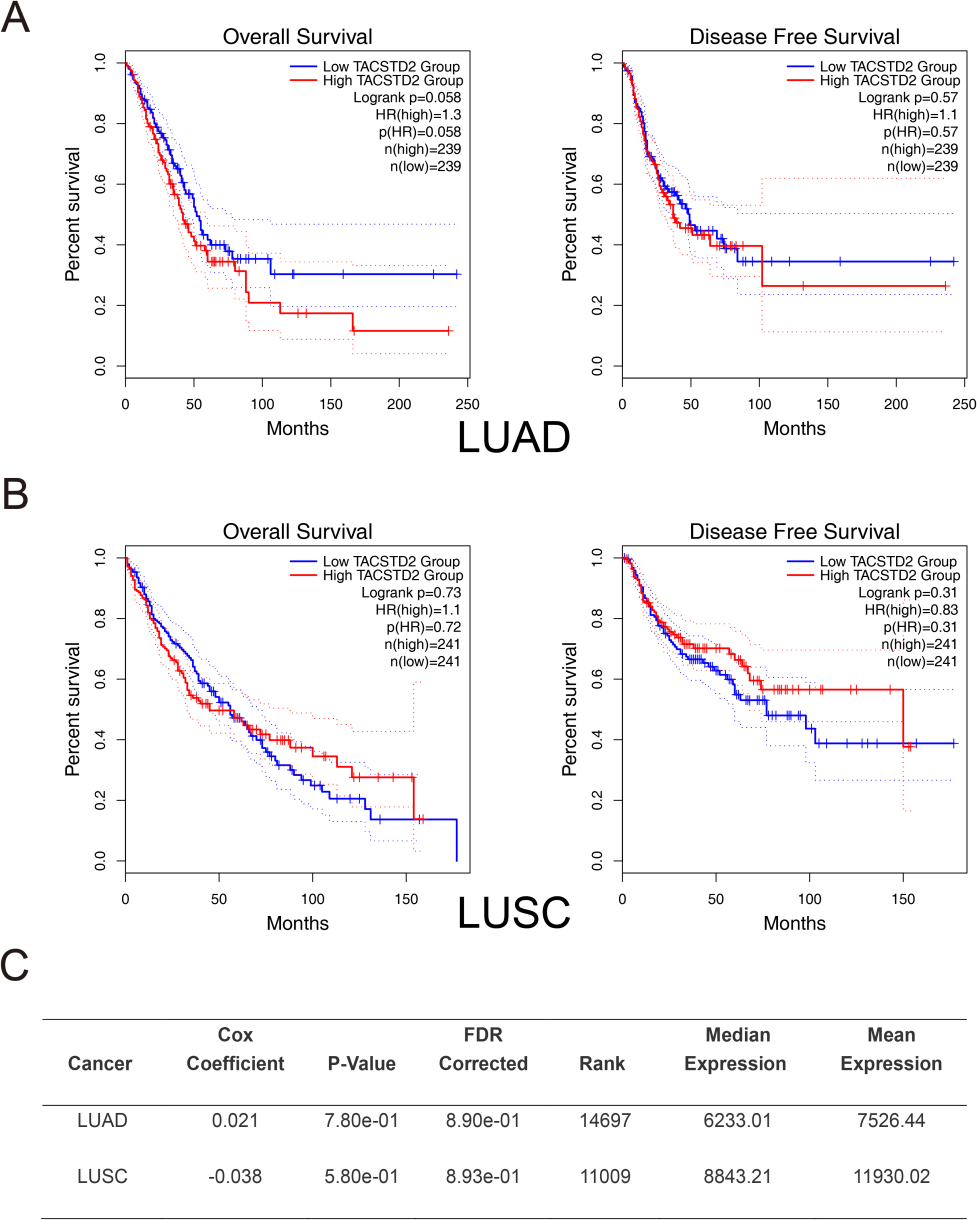
Preliminary Survival Analysis of TROP2 in Lung Cancer. Preliminary survival analyses were conducted using GEPIA2. **(A)** In LUAD, although high TROP2 expression trended towards a lower OS (P=0.058), the difference did not reach statistical significance. Disease-free survival (DFS) showed no association with TROP2 expression. **(B)** In LUSC, TROP2 expression was not significantly correlated with OS. Patients with high TROP2 expression trended toward better DFS, though this did not reach statistical significance. **(C)** Cox regression analysis via OncoLnc (http://www.oncolnc.org/) further confirmed the absence of a prognostic correlation for TROP2 in both LUAD and LUSC.

It may be even more complicated to identify the association of TROP2 with lung cancer prognosis, as shown in **Table 1**. Inamura K et al. found that TROP2 overexpression in adenocarcinoma was associated with higher lung cancer-specific mortality (hazard ratio (HR)=1.60, P=0.022) (41). Meanwhile, Mito R et al. found similar results, especially in cases with low differentiation and non-EGFR mutations (48). Conversely, Pak MG et al. reported opposing results: patients with high TROP2 expression—particularly those with stage II or III disease—had better OS and PFS (43). In HGNETs, both univariate and multivariate analyses showed that overexpressed TROP2 indicated a lower lung-cancer-specific mortality (41). In terms of squamous cell carcinoma, TROP2 might not be associated with patient mortality, as reported (41). These studies indicated that TROP2 exhibits heterogeneous associations with prognosis in lung cancers. We further performed survival analysis using bioinformatics methods including GEPIA and OncoLnc (http://www.oncolnc.org/) (44, 49, 50). Our results showed that the association of TROP2 with prognosis remains unclear, with trends observed but no statistical significance (**Figure 3**).

Notably, our preliminary survival analysis is also limited by the lack of protein-level data as discussed earlier. Meanwhile, our study also did not analyze the relationship between TROP2 expression and factors of the tumor microenvironment (TME), such as immune infiltration and tumor stemness. Both immune infiltration and tumor stemness play important roles in lung cancer and broadly affect prognosis (51–53). Therefore, future studies employing multi-level, multi-center, large-sample designs—incorporating subtype-specific stratified analyses and TME assessments—may better clarify the prognostic significance of TROP2 in lung cancer.

## 4 Molecular mechanisms of TROP2 in lung cancer development

### 4.1 Involvements in signaling pathways

**Figure 4:**
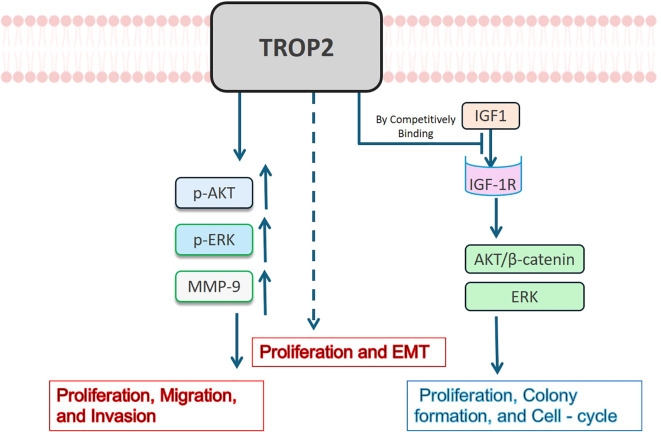
Mechanisms of TROP2 in LUAD. TROP2 has been reported to have contradictory effects on LUAD. It may enhance LUAD cell proliferation, migration, and invasion abilities via upregulating p-AKT, p-ERK, and MMP–9, and stimulate EMT through unknown mechanisms (shown in *red*). On the contrary, it has been reported to bind to IGF1 competitively, inhibiting the activation of AKT/β-catenin and ERK by IGF-1R, leading to proliferation and cell-cycle inhibition (shown in *blue*).

Given the unclear expression pattern of TROP2 in lung cancer, its functional effects and underlying mechanisms are likely complex. Li Z et al. manipulated TROP2 expression in A549 and PC9 cells and found it significantly enhanced the proliferation, migration, and invasion abilities of cancer cells (47). They further found that TROP2 significantly upregulated p-AKT, p-ERK, and MMP-9, suggesting it may promote the malignant phenotypes via the PI3K/Akt and MAPK pathways (47). Zheng WP et al. verified that Toxicarioside O could inhibit the proliferation and epithelial-mesenchymal transition (EMT) of A549 and H1299 cells by downregulating TROP2, which indirectly verifies its tumor-promoting effect (54). However, Lin JC et al. reported opposing effects: TROP2 significantly inhibited cell proliferation, colony formation, and cell-cycle progression in LUAD. They further demonstrated that TROP2 is competitively bound to IGF1, inhibiting the activation of AKT/β-catenin and ERK by IGF-1R, thus playing an anti-tumor role (42). All reported mechanisms of TROP2 in LUAD are summarized in **Figure 4**. These controversial effects suggest complex roles for TROP2 in LUAD. Extensive negative feedback mechanisms may lead to these contradictory findings. Clarifying the specific regulatory networks of TROP2 may identify breakthroughs to enhance the therapeutic efficacy and safety of ADC agents.

Moreover, besides the co-expression of TROP2 and p53 in squamous cell carcinoma reported by Mito R et al., the investigation of TROP2-related mechanisms in other types of lung cancer is extremely rare (48). Further exploration is needed in this area moving forward.

### 4.2 Role in TME remodeling and drug resistance

**Figure 5:**
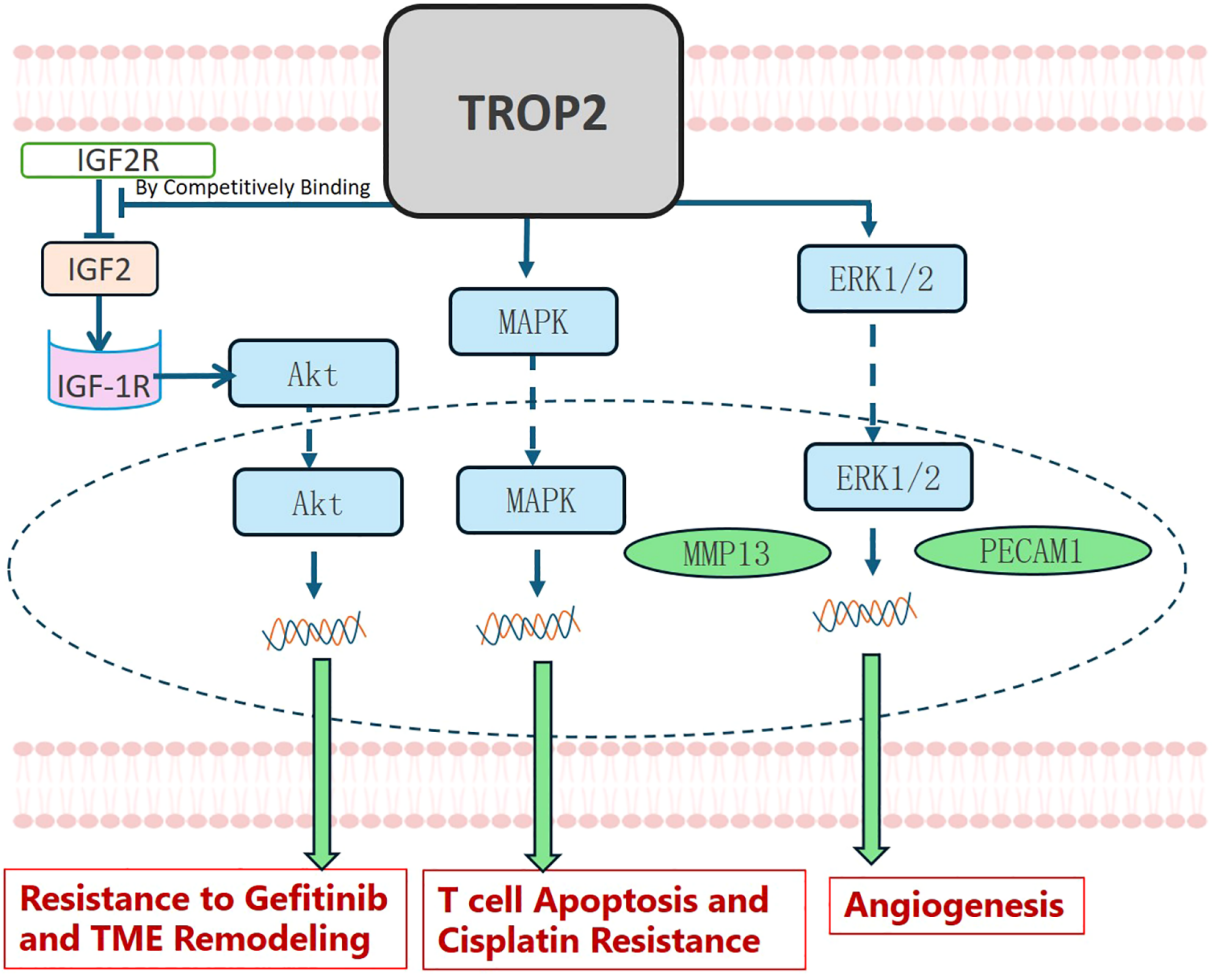
TROP2 in TME Remodeling and Drug Resistance. TROP2 promotes gefitinib resistance and TME remodeling by binding competitively to IGF2R, thereby activating the IGF2-IGF1R-Akt axis. It also stimulated T-cell apoptosis and cisplatin resistance via the MAPK signaling pathway. TROP2 further promoted angiogenesis by activating the ERK1/2 signaling pathway, leading to MMP13 and PECAM1 upregulation.

TROP2 may also be involved in TME remodeling and drug resistance in NSCLC, as shown in **Figure 5**. Guo X et al. verified that TROP2 promoted angiogenesis in NSCLC by activating the ERK1/2 signaling pathway and upregulating the expression of MMP13 and PECAM1 (55). Wang X et al. treated A549 and PC14 cells with cisplatin (DDP) and found it significantly promoted the expression of TROP2. They further demonstrated that TROP2 mediates T-cell apoptosis and DDP resistance via the MAPK signaling pathway (56). TROP2 may also promote EGFR-TKI resistance. Sun X et al. demonstrated that in NSCLC, TROP2 binds to IGF2R, promoting activation of the IGF2-IGF1R-Akt axis and thereby driving gefitinib resistance and TME remodeling (57).

## 5 Summary and future perspectives

Despite showing potential in lung cancer treatment, TROP2-targeted ADCs exhibit limited efficacy and a moderate safety profile. This is likely due to the complex interplay of TROP2’s variable expression patterns, prognosis-related heterogeneity, and intricate functional effects and regulatory mechanisms. Future research integrating interventional clinical studies (where researchers actively intervene and observe outcomes), observational clinical studies (where researchers observe without intervention), and basic studies, may overcome these challenges.

In observational clinical studies, identifying specific patient subgroups—analogous to how EGFR mutations predominate in Asian, female, and non-smoking adenocarcinoma patients—could improve treatment efficacy (58). Exploring novel molecular markers, such as co-expression patterns or gene co-mutations, enables the preselection of responsive patients, optimizing clinical application.

Basic research efforts should focus on elucidating the drivers of heterogeneity in TROP2 expression and determining whether such heterogeneity is temporally dynamic. These studies could provide insights into improving treatment safety. Investigating the regulatory mechanisms of TROP2—including potential negative feedback loops, external regulatory factors, and how these variables influence lung cancer progression—is also crucial. A comprehensive understanding of its expression regulation will significantly advance drug development.

In summary, TROP2 plays a multifaceted role in lung cancer. Integrating interventional clinical, observational and basic research is essential to improve the efficacy and safety of TROP2-targeted ADCs for lung cancer.

## Glossary

TACSTD2: Tumor-associated calcium signal transducer 2
TROP2: Trophoblast cell-surface antigen 2 (an alias of TACSTD2)
ADCs: Antibody-drug conjugates
NSCLC: Non-small cell lung cancer
SCLC: Small cell lung cancer
PDX: Patient-derived xenograft
Dato-DXd: Datopotamab deruxtecan
IHC: Immunohistochemistry
SG: Sacituzumab govitecan
TNBC: Triple-negative breast cancer
mUC: Metastatic urothelial cancer
EGFR: Epidermal growth factor receptor
HGNET: High-grade neuroendocrine tumors
LOH: Loss of heterozygosity
GEPIA: Gene Expression Profiling Interactive Analysis
TCGA: The Cancer Genome Atlas
LUAD: Lung adenocarcinoma
LUSC: Lung squamous cell carcinoma
DEGs: Differentially expressed genes
HR: Hazard ratio
OS: Overall survival
PFS: Progression-free survival
TME: Tumor microenvironment
EMT: Epithelial-mesenchymal transition
DDP: Cisplatin
DFS: Disease-free survival.
